# A Rare Case of Kartagener Syndrome Presenting with Sinusitis, Situs Inversus, and Bronchiectasis: Emphasizing Early Diagnosis and Management Strategies

**DOI:** 10.7759/cureus.41890

**Published:** 2023-07-14

**Authors:** Samia Rauf R Butt, Hassan Shakoor, Tayyaba J Khan, Bsher Almaalouli, Chukwuyem Ekhator, Safa Ansari, Nehal Shaikh, Abdullah Shehryar, Abdur Rehman

**Affiliations:** 1 General Practice, California Institute of Behavioral Neurosciences & Psychology, Fairfield, USA; 2 Internal Medicine, Fauji Foundation Hospital Islamabad, Islamabad, PAK; 3 Medicine, Liaquat University of Medical and Health Sciences, Jamshoro, PAK; 4 Faculty of Medicine, Damascus University, Damascus, SYR; 5 Neuro-Oncology, New York Institute of Technology, College of Osteopathic Medicine, Old Westbury, USA; 6 Medicine and Surgery, Karachi Medical and Dental College, Karachi, PAK; 7 Department of Medicine, Ghulam Muhammad Mahar Medical College, Sukkur, PAK; 8 Internal Medicine, Allama Iqbal Medical College, Lahore, PAK; 9 Surgery, Mayo Hospital, Lahore, PAK

**Keywords:** supportive pulmonary care, chronic rhinosinusitis with nasal polyps, chronic sinusitis, bronchiectasis exacerbation, situs inversus with dextrocardia, primary ciliary dyskinesia (pcd), kartagener syndrome (ks)

## Abstract

Primary ciliary dyskinesia (PCDs), a subset of ciliary motility disorders, includes the rare hereditary illness Kartagener syndrome (KS). Sinusitis, situs inversus, and bronchiectasis, brought on by aberrant ciliary activity, are its defining features. We describe a case of an 18-year-old female with a history of recurrent respiratory complaints and chronic sinusitis. Additional testing confirmed the diagnosis of KS by identifying situs inversus, chronic bronchiectasis, and nasal polyps. This instance emphasizes the value of prompt KS diagnosis and treatment to avoid consequences. Supportive pulmonary care, antibiotics, and chest physical therapy are frequently employed, despite the lack of therapeutic standards. To further understand and manage this illness, more research is required. Patients with recurrent respiratory infections and structural lung disease can identify KS early.

## Introduction

Kartagener syndrome (KS) is a type of primary ciliary dyskinesia (PCDs), a group of ciliary motility disorders [1]. It is a genetic condition characterized by sinusitis, situs inversus, and bronchiectasis due to abnormal ciliary movement [2]. The connection between situs inversus, chronic sinusitis, and bronchiectasis was first described by Siewert in 1904 [2]. Later in 1933, Kartagener identified this congenital syndrome and named it after himself [2]. It affects around one in 30,000 live births [3].

Normal ciliary activity is vital for sperm motility, respiratory defense, and proper embryonic visceral orientation. KS, caused by mutations in the DNAI1 and DNAH5 genes, reduces ciliary motility, leading to infertility, recurrent sinopulmonary infections, and left-right body orientation difficulties [4].

Diagnosis is sometimes suspected prenatally if situs inversus is detected on obstetric ultrasound. However, most cases are identified in childhood after a series of respiratory infections. Doctors may notice right hemithorax heart sounds during the examination, and imaging tests like chest X-rays and scans of the abdomen and paranasal sinuses may reveal dextrocardia. Symptomatic treatment involves using antibiotics to manage associated infections [5].

## Case presentation

An 18-year-old female presented to the pulmonology outpatient department with a 10-year history of headaches. She also reported shortness of breath and decreased sense of smell for the past year. The patient experienced gradual onset of moderate-intensity headaches localized in the frontal and bilateral temporal regions since the age of eight. Headaches worsened in the prostration position and were persistent with no notable relieving factors. There were no associated symptoms of vertigo, altered state of consciousness, or seizures. The patient experienced episodes of rhinorrhea and epiphora, which resolved spontaneously after a variable duration of time. A diagnosis of chronic sinusitis was made 10 years ago, and the patient was initially prescribed topical steroids and oral antibiotics without significant improvement. Subsequently, functional endoscopic sinus surgery (FESS) was performed, revealing situs inversus during preoperative assessment. The surgery provided temporary relief, but symptoms recurred after one year. However, symptoms were alleviated with the use of topical steroids and decongestants.

The patient's previously described symptoms worsened despite topical steroids and oral antibiotics. New symptoms included persistent shortness of breath (SOB) and decreased sense of smell for one year. SOB worsened during sleep. No history of exertional SOB, orthopnea, paroxysmal nocturnal dyspnea, wheezing, chest pain, hemoptysis, fever, or weight loss was reported.

The patient's past history includes a surgical intervention performed 10 years ago, as previously mentioned. No significant allergies were reported. She was born to parents who were blood relatives (consanguineous couple). There were no siblings to the patient. In the family history, her father had a history of tuberculosis, which was successfully treated with antitubercular therapy.

General examination revealed no remarkable findings, including nail clubbing. Vitals were normal. Nasal rhinoscopy showed a right deviated nasal septum, erythematous and edematous nasal mucosa, and nasal polyps. Respiratory examination revealed decreased breath sounds on the left side compared to the right side. Inspiratory crackles were heard at the base of the right lung and left lower axillary region. Systemic examination of other systems was unremarkable.

**Figure 1:**
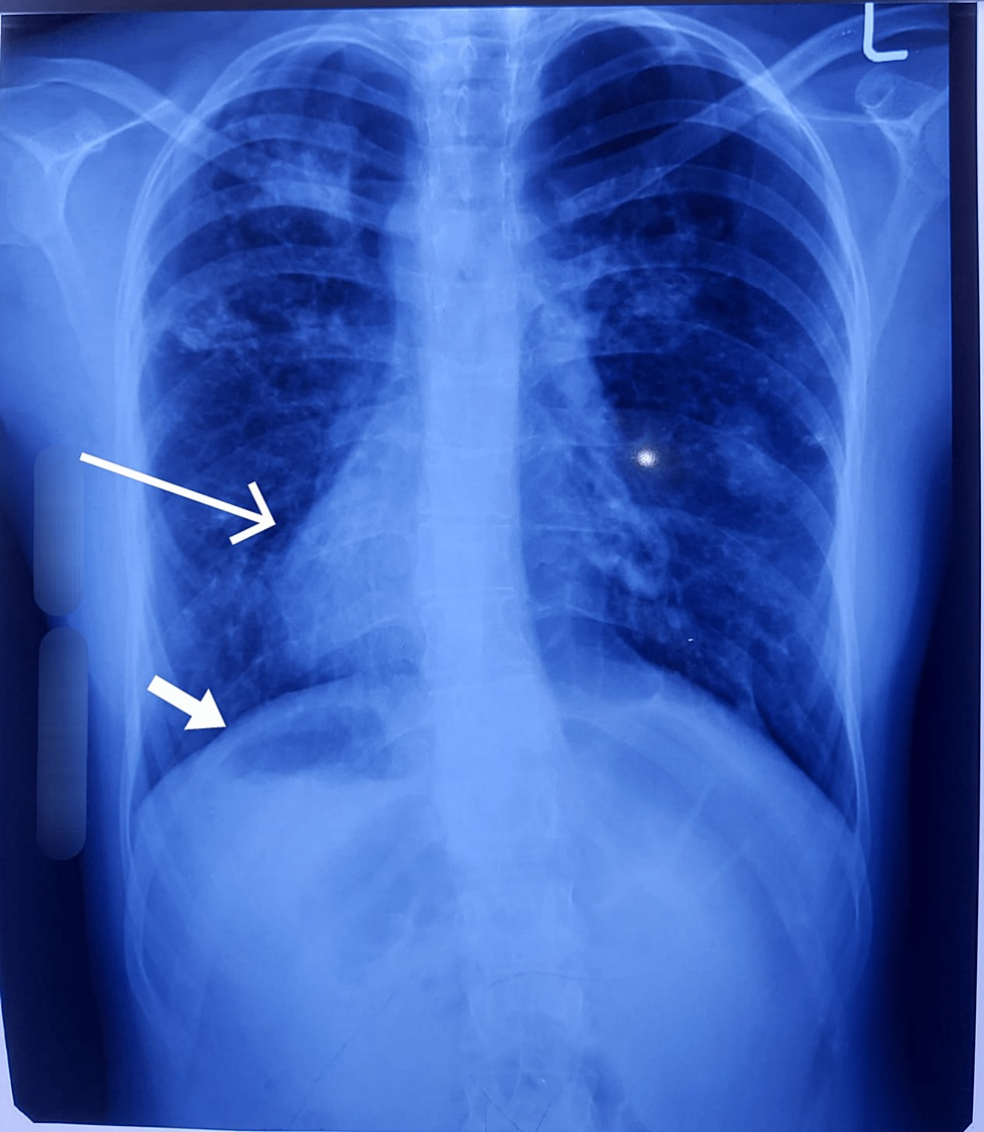
Chest X-ray: long arrow showing dextrocardia, short arrow showing gastric bubble on right side. Bilateral bronchovascular marking prominent in lower lobes, consistent with bronchiectasis.

Complete blood workup was performed. The details are provided in Table 1. Only mild leukocytosis was found. Chest X-ray was also performed. It showed situs inversus with dextrocardia, liver dullness on the left side and gastric bubble on the right side. Chest X-ray is shown in Figure 1.

**Table 1:** Complete blood workup revealing leukocytosis INR: international normalized ratio, APTT: activated partial thromboplastin time, WBC count: white blood cells count, RBC: red blood cells, HCT: hematocrit, MCV: mean corpuscular volume, MCH: mean corpuscular hemoglobin, MCHC: mean corpuscular hemoglobin concentration, ALT: alanine transaminase, AST: aspartate aminotransferase, ALP: alkaline phosphatase

Coagulation Profile		
		Reference range
Prothrombin Time-Control	13	10-14 seconds
Prothrombin Time-Patient	13	Up to 13 seconds
INR	1.0	0.9-1.3
Control Time	28	25-35 seconds
APTT	26	Up to 31 seconds
Hemogram		
WBC count	13.2	4-11 x10^9^/L
Total RBC	4.3	3.8-5.2 x10^12^/l
Hemoglobin	12	13-18 (g/dL)
HCT	39	35-46%
MCV	89	77-95 fl
MCH	28	26-32 (pg)
MCHC	31	32-36 (g/dL)
Platelets	303	150-400 x10^9^/L
Neutrophils	60.1	40-80%
Lymphocytes	31.7	20-40%
Monocytes	6.6	2-10%
Eosinophils	0.8	1-6%
Renal Function Tests		
Urea	16.46	10-50 mg/dl
Serum Creatinine	0.58	0.5-0.9 mg/dl
Liver Function Tests		
Bilirubin total	0.5	0.3-1.2 mg/dl
Total protein	6.3	5.7-8.2 g/dl
Albumin	4.0	3.2-4.8 g/dl
ALT	14	Up to 40 U/L
AST	15	Up to 40 U/L
ALP	82	40-120 U/L
Serum Electrolytes		
Sodium	139	135-145 mmol/L
Potassium	5.73	3.5-5 mmol/L
Chloride	103	98-107 mmol/L
Calcium	9.1	8.5-10.5 mg/dl

**Figure 2:**
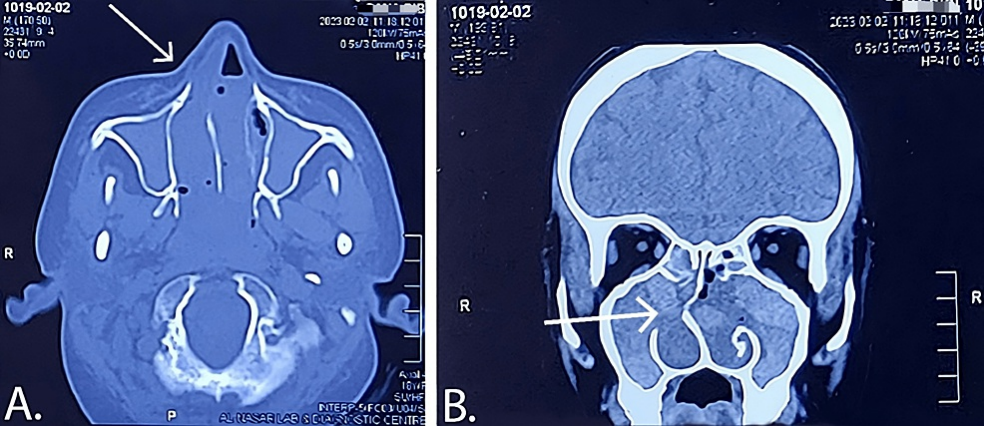
CT scan paranasal sinuses. Panel A shows axial view, panel B shows coronal view. The ethmoid, maxillary, and sphenoid sinuses are opaque bilaterally, with low-density soft tissue mass. Both nasal cavities are also opaque, with similar soft tissue mass, as marked by arrow in panel A. Deviation of nasal septum to right is noted, as marked by arrow in panel B.

A CT scan of the paranasal sinuses revealed bilateral opacities with low-density soft tissue material in the ethmoid, sphenoid, and maxillary sinuses. The nasal cavities also exhibited similar opacities. The left frontal sinus was non-pneumatized. Sino-nasal polyposis was diagnosed based on these findings. A CT paranasal sinuses image is described in Figure 2. Considering the patient's history, examination, and investigation, chronic sinusitis was confirmed.

**Figure 3:**
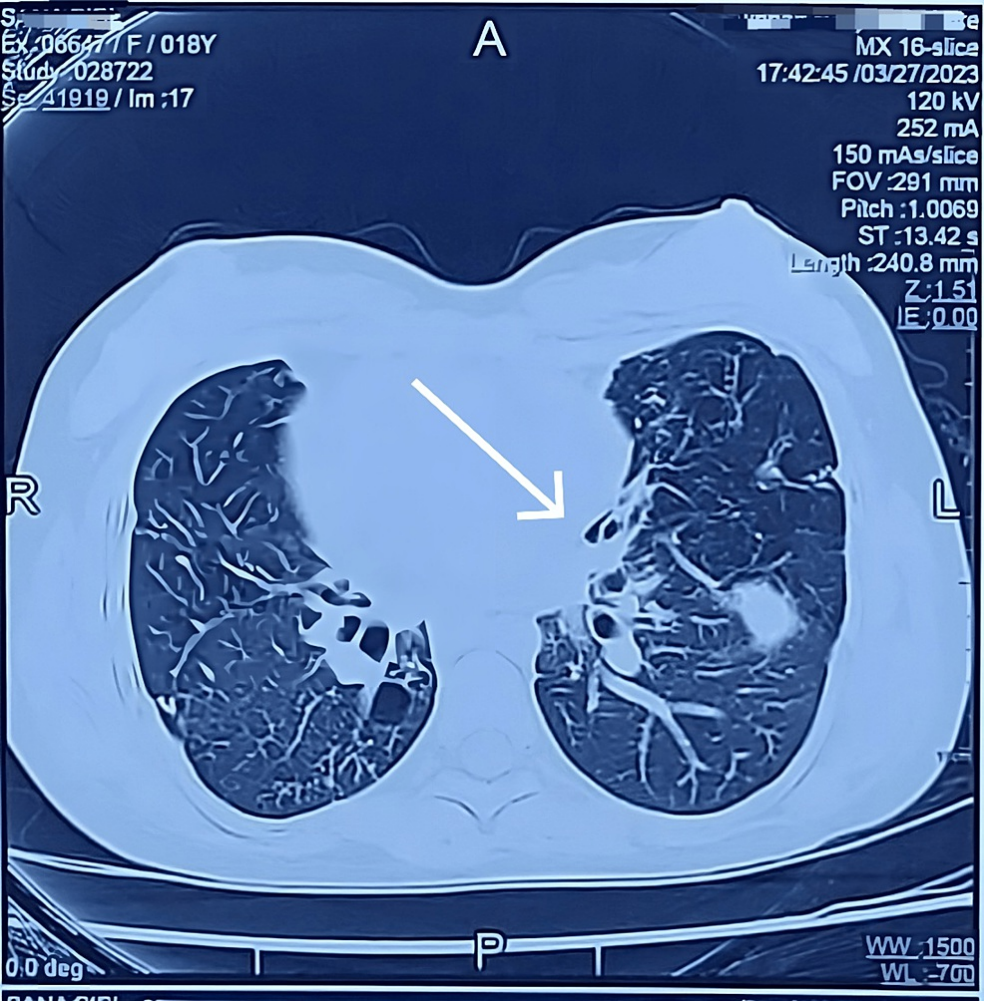
CT scan chest: image reveals bronchiectasis with involvement of the left side more than the right side, as shown by the arrow. Bronchoceles can also be appreciated.

For the lower respiratory tract, initially spirometry was performed which pointed toward restrictive lung disease. Hence high-resolution computed tomography (HRCT) scan of the lungs was performed. CT scan confirmed bronchiectasis affecting lower lobes with involvement of the left side more than the right side. CT scan chest is shown in Figure 3. Based on the findings, bronchiectasis was confirmed.

History, clinical signs, and imaging studies proved the diagnosis of Kartagener syndrome. Antibiotics and chest physiotherapy were advised. A regimen of oral azithromycin with cetirizine, topical steroids, nebulization with hypertonic solution and nasal decongestants was planned for the patient. Patient followed up a week later with improvement in symptoms. Patient was also referred to otorhinolaryngology consult, which decided close monitoring of patient status with a medical approach before embarking on a surgical approach. The nature of her disease and importance of regular follow-up was explained to patient.

## Discussion

Ciliary motility disorders can be inherited or acquired. Congenital conditions are known as primary ciliary dyskinesia (PCDs), with situs inversus present in about 50% of PCD patients [2]. Immotile-cilia syndrome is an uncommon, autosomal recessive hereditary condition that affects the function of cilia in the fallopian tube and respiratory system. It leads to chronic recurrent pneumonia, bronchiectasis, otitis media, and rhinosinusitis caused by pseudomonal infection. When situs inversus, chronic sinusitis, and bronchiectasis co-occur, it is referred to as Kartagener syndrome [1].

Clinical signs and symptoms of PCD vary. Some individuals may experience neonatal respiratory distress that progresses to chronic productive cough due to bronchiectasis. Other symptoms include non-responsive atypical asthma, chronic rhinosinusitis, otitis, ectopic pregnancy, subfertility in women, or male infertility [6]. As the condition worsens, bronchiectasis becomes clinically evident on radiographs. In preschool-age children, bronchiectasis and obstructive impairment may be observed [7].

Diagnosis of cilia dysfunction involves genetic analyses, biopsies, and tests [8]. Specialized labs can genetically test for mutations in the DNAI1 and DNAH5 genes. The presence of bi-allelic mutations confirms the diagnosis, while the identification of a trans-allelic mutation indicates the presence of a single allelic mutation [9]. The diagnostic criteria for Kartagener syndrome include a history of chronic bronchial infection and rhinitis from early childhood, along with one or more of the following features: (i) situs inversus or dextrocardia in the patient or a sibling, (ii) immotile spermatozoa, (iii) impaired tracheobronchial clearance, and (iv) cilia displaying characteristic ultrastructural defects on electron microscopy [3]. These criteria were satisfied in our case, leading to the diagnosis of KS.

When considering a potential diagnosis of Kartagener's syndrome, other conditions should be considered, such as adenoid hyperplasia, allergic bronchopulmonary aspergillosis, alpha1-antitrypsin (AAT) deficiency, bronchial obstruction, chronic aspiration, chronic obstructive pulmonary disease (COPD), congenital cartilage deficiency, cystic fibrosis, foreign body aspiration, idiopathic interstitial pneumonia, and idiopathic nasal polyposis. However, in this case, the patient's medical history revealed chronic sinusitis with polyposis, which was confirmed by further investigations. Additionally, the presence of situs inversus provided a significant clue supporting the inclusion of Kartagener's syndrome as the primary consideration among the differential diagnoses.

Different diagnoses should be considered depending on when the symptoms first appear. In newborns, transient tachypnea is common, while in children and adults, other conditions such as cystic fibrosis, chronic granulomatous diseases, humoral immunodeficiencies, allergic bronchopulmonary aspergillosis, vasculitis, severe asthma, and allergic rhinitis with unusual chronic sinusitis can also cause similar symptoms [5].

Treatment for Kartagener syndrome involves supportive pulmonary care, antibiotics with excellent pseudomonal coverage, and daily chest physiotherapy. The efficacy of DNase and other mucolytic medications, such as hypertonic saline and acetylcysteine, has not been extensively studied but may be explored, particularly in patients with recurring infections or persistent respiratory symptoms [1].

## Conclusions

Early diagnosis is crucial for effectively managing Kartagener's syndrome (KS) and preventing complications. While cystic fibrosis and KS are commonly identified in early infancy, KS can occasionally remain asymptomatic for extended periods. Timely detection of KS is essential for a better prognosis and symptom control, as respiratory problems in KS patients stem from cilia abnormalities that can cause organ mal-positioning and structural changes. Therefore, when a patient presents with recurrent respiratory infections and structural lung abnormalities, consideration should be given to primary ciliary dyskinesia (PCD) as an underlying cause. However, the scarcity of major randomized control trials in KS has led to a lack of established management guidelines. By addressing this research gap, healthcare providers can improve their understanding of Kartagener syndrome and develop targeted approaches for optimal management, ultimately enhancing the quality of care provided to individuals affected by this condition.
